# Supplementary material for: Evidence supported by Mendelian randomization: impact on inflammatory factors in knee osteoarthritis
Source: Front Med (Lausanne). 2024 May 28;11:1382836. doi: 10.3389/fmed.2024.1382836 (PMC11165061; doi:10.3389/fmed.2024.1382836)
Supplement: Supplementary file 1 [file Data_Sheet_1.ZIP › data/GCST90274837_5e6/837.leaveoneout.pdf]

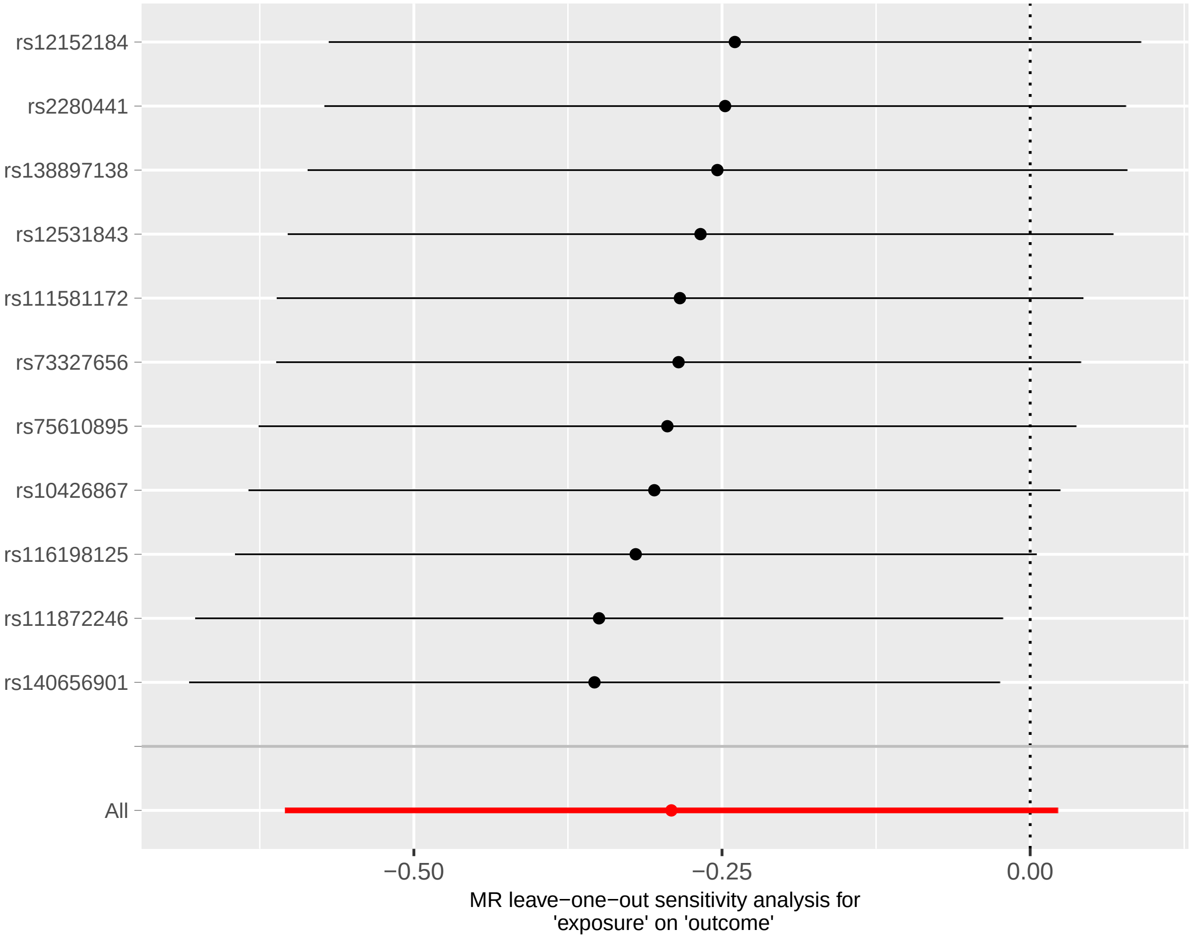
rs12152184
rs2280441
rs138897138
rs12531843
rs111581172
rs73327656
rs75610895
rs10426867
rs116198125
rs111872246
rs140656901
All
−0.50
−0.25
0.00
MR leave−one−out sensitivity analysis for
'exposure' on 'outcome'
